# Mirror‐Image Packing Provides a Molecular Basis for the Nanomolar Equipotency of Enantiomers of an Experimental Herbicide

**DOI:** 10.1002/anie.201607185

**Published:** 2016-09-26

**Authors:** Claudine Bisson, K. Linda Britton, Svetlana E. Sedelnikova, H. Fiona Rodgers, Thomas C. Eadsforth, Russell C. Viner, Tim R. Hawkes, Patrick J. Baker, David W. Rice

**Affiliations:** ^1^ Krebs Institute for Biomolecular Research, Department of Molecular Biology and BiotechnologyUniversity of SheffieldFirth CourtWestern BankSheffieldS10 2TNUK; ^2^ Division of Biological Chemistry and Drug DiscoveryWellcome Trust BiocentreUniversity of DundeeDundeeDD1 5EHUK; ^3^ Syngenta, Jealott's Hill International Research StationBracknellRG42 6EYUK

**Keywords:** chirality, drug design, enantioselectivity, inhibitors, structural biology

## Abstract

Programs of drug discovery generally exploit one enantiomer of a chiral compound for lead development following the principle that enantiomer recognition is central to biological specificity. However, chiral promiscuity has been identified for a number of enzyme families, which have shown that mirror‐image packing can enable opposite enantiomers to be accommodated in an enzyme's active site. Reported here is a series of crystallographic studies of complexes between an enzyme and a potent experimental herbicide whose chiral center forms an essential part of the inhibitor pharmacophore. Initial studies with a racemate at 1.85 Å resolution failed to identify the chirality of the bound inhibitor, however, by extending the resolution to 1.1 Å and by analyzing high‐resolution complexes with the enantiopure compounds, we determined that both enantiomers make equivalent pseudosymmetric interactions in the active site, thus mimicking an achiral reaction intermediate.

The chiral nature of the active site of proteins generally gives rise to the differential recognition of chiral substrates and inhibitors. As a result, during programs of drug development, an enzyme's stereochemical preferences can be exploited to achieve target selectivity and reduce the risk of toxicity.^2^ Although a chirally pure compound is often the desired product of such schemes, the early stages of both pharmaceutical and agrochemical inhibitor development typically start from synthetic racemates, which are often easier and more cost effective to synthesize than a specific enantiomer (see Ref. 2, 3 and references therein). The program of herbicide development described herein aims to identify inhibitors of imidazoleglycerolphosphate‐dehydratase (IGPD; EC 4.2.1.19), an enzyme of histidine biosynthesis in plants and microorganisms. This enzyme catalyzes the manganese(II)‐dependent dehydration of (2*R*,3*S*)‐2,3‐dihydroxy‐3‐(1*H*‐imidazol‐5‐yl)propyl dihydrogen phosphate (2*R*,3*S*‐IGP) to 3‐(1*H*‐Imidazol‐4‐yl)‐2‐oxopropyl dihydrogen phosphate (IAP; Figure 1).^4^ IGPD shows strict enantioselectivity for its substrate, with the other three diastereoisomers of IGP acting as competitive inhibitors of the enzyme,^5^ thus indicating that the active site shows some flexibility to accommodate inverted chiral centers. Previous studies have shown that IGPD is inhibited by triazolylphosphonates, whose potency is based on mimicking key reaction intermediates.^6^ In this paper we describe work on the lead compound, 2‐hydroxy‐3‐(1,2,4‐triazol‐1‐yl) propylphosphonate (**C348**; Figure 1) and present a series of structures of IGPD, from *Arabidopsis thaliana*, complexed with a synthetic racemate of **C348** and with the enantiopure compounds. The structural data show that both the (*R*)‐ and (*S*)‐**C348** make equivalent interactions with the active site of IGPD and bind with mirror‐image packing. Data from both in vivo and in vitro assays further shows that both enantiomers are equipotent nanomolar inhibitors of the enzyme. By comparing our enzyme/inhibitor complexes with that of structures of IGPD complexed with the substrate, 2*R*,3*S*‐IGP, we provide a molecular explanation of this finding.

**Figure 1:**
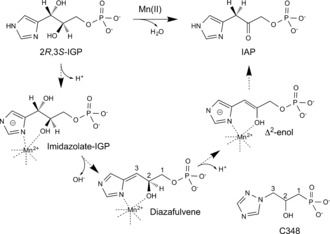
A schematic diagram outlining the reaction mechanism of IGPD and showing the chemical structure of **C348**. Backbone carbon atoms of **C348** and the diazafulvene intermediate are numbered 1–3. The chiral center of **C348** is at C2.

Crystals of a ΔN construct of IGPD isoform 2 from *Arabidopsis thaliana* (*At* ΔN IGPD2 construct A; see Supporting Information for experimental methods) were grown in the presence of a racemate of **C348** and the structure of the enzyme/inhibitor complex was determined to 1.85 Å resolution. The resulting electron density showed clear evidence for the ordering of the C‐loop of IGPD2 (residues 193–206; see Figure S1 in the Supporting Information), with the enzyme adopting the same closed conformation as seen in the previously determined structure of an inactive mutant (E21Q) of *At* IGPD2 with its substrate, IGP (PDB: 4MU4).^7^ Difference density for **C348** could be identified within the active site and the triazole ring could be modelled between the two manganese ions with the N2 and N4 atoms forming ligands to Mn1 and Mn2, respectively. The **C348** C2−OH group acts as an additional ligand to Mn1 and the phosphonate group is bound in a positively charged pocket, surrounded by the side chains of R99, R121, K177, S199, and K201, and by water‐mediated hydrogen bonds to Q51 and H55. However, despite the 1.85 Å resolution of the data, there was a lack of electron density around C3 of the inhibitor (see Figure S2a). And, as a result, whilst the major functional groups of the inhibitor could be identified, the chirality of the bound ligand was uncertain.

A shorter ΔN construct of IGPD2 (*At* ΔN IGPD2 construct B) yielded better quality diffracting crystals and produced a structure of the IGPD2/**C348** complex at 1.1 Å resolution (PDB: 5EKW; Table 1). However, as with the electron density for **C348** in the 1.85 Å structure, the density at 1.1 Å resolution was also weak around C3. Nevertheless, a small peak could be observed in the map around C3 when contoured at 1 σ, the position of which was consistent with the binding of the (*S*)‐**C348** (see Figure S2b). Refinement of this structure using tight geometric restraints revealed further difference features, indicating a second position for the inhibitor, which, from the geometry, could only be interpreted as resulting from the binding of the (*R*)‐**C348** (see Figure S2c,d). This finding indicated that in the crystal, the active sites of some of the enzyme molecules were occupied by (*S*)‐**C348** and others were occupied by (*R*)‐**C348**. Subsequent refinement of this structure with occupancies of 0.6 and 0.4 for the (*S*)‐ and (*R*)‐**C348**, respectively, produced a model which fully explained the electron density, thus indicating that both enantiomers of **C348** were bound to an essentially invariant enzyme structure.

**Table 1:** Data collection and refinement statistics.

	*At* IGPD2 + racemate **C348** PDB: 5EKW	*At* IGPD2 + (*R*)‐**C348** PDB: 5ELW	*At* IGPD2 + (*S*)‐**C348** PDB: 5EL9	*Pf* IGPD + (*R*)‐**C348** PDB: 5DNX	*Pf* IGPD + (*S*)‐**C348** PDB: 5DNL
*Data collection*:					
Wavelength [Å]	0.95070	0.98020	0.97960	0.9794	0.9794
Space group	P432	P432	P432	I422	I422
Cell dimensions; *a*, *b*, *c* [Å]	112.9, 112.9, 112.9	112.6, 112.6, 112.6	112.6, 112.6, 112.6	140.4, 140.4, 136.7	141.3, 141.3, 137.4
Resolution [Å]	35.7–1.1 (1.12–1.1)	45.96–1.36 (1.4–1.36)	45.98–1.1 (1.13–1.1)	48.98–1.8 (1.85–1.8)	49.25–1.53 (1.57–1.53)
Total observations^[c]^	1 221 822 (144 649)	474 417 (25 277)	1 625 442 (48 528)	1 030 031 (74 677)	284 672 (34 864)
Unique observations^[c]^	99 289 (14 237)	52 156 (3369)	98 751 (6973)	63 077 (4586)	54 525 (7257)
*R*_merge_^[a,c]^	0.082 (0.676)	0.068 (0.66)	0.063 (0.650)	0.118 (1.006)	0.080 (0.688)
*R_pim_* ^[b,c]^	0.025 (0.175)	0.026 (0.271)	0.017 (0.275)	0.030 (0.257)	0.039 (0.141)
*I*/σ*I* ^[c]^	15.1 (2.2)	23.8 (2.8)	28.0 (2.9)	22.1 (4.6)	18.1 (4.1)
Completeness (%)^[c]^	99.5 (98.3)	99 (89.1)	99.6 (96.8)	100 (100)	100 (100)
Redundancy^[c]^	10.6 (5.1)	9.1 (7.5)	16.5 (7.0)	16.3 (16.3)	13.1 (13.2)
*Refinement*:					
*R*_work_/*R*_free_	12.3/13.7	11.7/13.1	11.4/12.9	13.1/15.8	13.5/14.9
*No. Atoms*:					
Protein	1569	1554	1607	4195	4196
Ligand/ions	78	32	52	45	45
Water	235	204	207	322	476
*B‐factors*:					
Protein	13.5	10.3	11.7	22.2	19.0
Ligand/ions	13.0	17.2	12.0	15.0	12.35
Water	26.8	23.1	27.5	29.0	27.6
*RMS deviations*:					
Bond lengths [Å]	0.01	0.01	0.01	0.01	0.09
Bond angles [°]	1.79	1.59	1.73	1.51	1.47

[a] *R*_merge_=Σ_*hkl*_Σ_*i*_|*I_i_*−*I_m_*|/Σ_*hkl*_Σ_*i*_ *I_i_*. [b] *R*_pim_=Σ_*hkl*_/n−1Σ_*i*=1_|*I_i_*−*I_m_*|/Σ_*hkl*_Σ_*i*_ *I_i_*, where *I_i_* and *I_m_* are the observed intensity and mean intensity of related reflections, respectively. [c] Values within parentheses are for data in the high‐resolution shell.

To confirm that both enantiomers of **C348** bind to IGPD2, and to improve our interpretation of the mixed structure, we resolved the **C348** racemate by HPLC to greater than 98 % enantiopurity. The binding affinities for the enantiopure compounds, (*S*)‐ and (*R*)‐**C348**, against *At* ΔN IGPD2 (construct B) were measured by an in vitro enzyme assay and gave apparent *K*_i_ values of (25±3) and (15±5) nm, respectively (Figure 2 a; see Figure S3). Glasshouse spray studies, comparing the herbicidal properties of the two enantiomers and the racemate, showed they all exhibited similar efficacy in terms of the spray rate (kg/Ha) necessary to cause 50 % damage (as visually assessed; see Figure S4). Whilst we had initially expected that the two enantiomers would have quite different binding affinities and herbicidal activity, they were found to be equipotent inhibitors of IGPD2. The enantiopure compounds were then co‐crystallized with *At* IGPD2 ΔN construct B to produce structures at 1.15 Å and 1.5 Å resolution, for the *S* (PDB: 5EL9) and *R* forms (PDB: 5ELW), respectively. The conformation of the enzyme and the position of the metal ions were equivalent in each complex, with the only clear difference, apart from the inhibitor conformation, being minor changes in the solvent structure within the active site (see Figure S5). In both structures high quality electron density covered all the atoms of the inhibitor (Figure 2 b,c), thus confirming that the additional difference features seen in the complexes with the racemate arose as a result of mixed binding. The resulting models show that for each chiral form of **C348**, the positions of the phosphonate group, N2 and N4 of the triazole ring, and the C2−OH substituent superimpose almost exactly and make equivalent interactions within the active site of the enzyme (Figure 2 d; see Figure S6a). This arrangement is achieved, despite the inversion in chirality, by a combination of torsion‐angle changes around the C3−C2 and C2−C1 bonds of the inhibitor, together with a difference in the tilt of the triazole ring around the Mn1–Mn2 vector. The two enantiomers thus trace an inverted path between the metal binding site and the phosphonate binding site, whereby they are related by mirror‐image packing (Figure 3 a,b). Both enantiomers retain low‐energy conformations with approximately staggered torsion angles, with the largest deviation in the atomic position of equivalent atoms occurring at C3 of the backbone, thus explaining the weak density at this position observed in the complexes of IGPD2 with the racemate.

**Figure 2:**
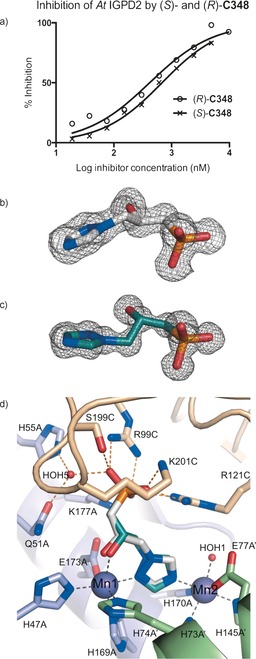
Both enantiomers of **C348** are equipotent inhibitors of IGPD2. a) In vitro inhibition data for (*R*)‐**C348** (open circles) and (*S*)‐**C348** (crosses) fit to log IC_50_ values of 2.62 and 2.84 (95 % confidence limits ± log 0.15, Hill coefficients of ≈0.8), respectively, and correspond to *K*_i_ values of about (14±4) and (23±3) nm. Fitting was carried out by nonlinear regression with equal weighting of data points using GraphPad Prism (see Figure S3). Fo‐Fc omit maps (gray mesh) for the 1.4 Å resolution (*R*)‐**C348** (white carbon atoms)/IGPD2 complex structure (b) and the 1.1 Å resolution (*S*)‐**C348** (teal carbon atoms)/IGPD2 complex structure (c) contoured at 3 σ. d) A superposition of the structures of the two **C348** enantiomers in complex with IGPD2, thus showing how the active site can accommodate both enantiomers of **C348**. A stereo view of the same image is provided in Figure S6a.

**Figure 3:**
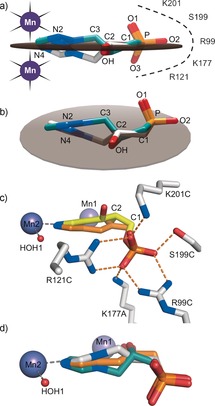
The equivalent planar arrangement of substituent groups in (*R*)‐ and (*S*)‐**C348** generates a pseudo‐mirror plane. a,b) The plane (gray disk) was calculated in Chimera^1^ based on the average positions of the N2, N4, C2−OH, and C1−P bond positions for both enantiomers of **C348**, which are colored teal and white for the for the *S* and *R* enantiomers, respectively. c) A model of the Δ^2^‐enol (orange) in the imidazole‐IGP (yellow)/IGPD2 complex (PDB: 4MU4). The sp^2^ C2 of the enol can be accommodated without altering the position of the phosphate group. d) The modelled Δ^2^‐enol bisects the positions of the two enantiomers of **C348**.

Mirror‐image packing of opposite enantiomers of ligands to enzymes and receptors has been reported previously. Examples include the binding of d/l‐phenylalanine and the superinhibitors d/l‐2‐aminooxy‐3‐phenylpropionic acid (d/l‐AOPP) to phenylalanine ammonia‐lyase,^8^ d/l‐malate to citrate synthase,^9^ and d/l‐isocitrate to isocitrate dehydrogenase,^10^ amongst others (see Figure S7).^11^ In each of these examples, and in the binding of the two enantiomers of **C348** to IGPD, examination of the structures shows that the position of three R groups around the chiral center is approximately maintained on the enzyme surface by flipping the direction of the hydrogen atom at the fourth position, by 180°, after inversion of chirality. This corresponds to the approach of the free ligand to the enzyme surface in an inverted configuration and generates a small separation in the position of the chiral center of each enantiomer on either side of the pseudo‐mirror plane. Subtle changes to the dihedral angles formed in the pendant groups optimize the fit within the chiral environment of the active site. On a case‐by‐case basis, whether or not such a situation is feasible depends on there being sufficient space to accommodate the two positions of the chiral center and the new position of the fourth ligand forming the chiral group (commonly a hydrogen atom). In addition, the chemical nature of the pendant groups dictates whether changes in the dihedral angles are energetically accessible. Any small differences in geometry resulting from the optimization of the fit to the active site will necessarily give rise to a difference in the affinity of the two enantiomers, but, as we show in IGPD, such differences can be remarkably small and both enantiomers can act as highly potent inhibitors.

As both enantiomers of **C348** bind to *At* IGPD2, high‐resolution structures of *Pyrococcus furiosus (Pf)* IGPD (38 % sequence identity to *At* IGPD2) complexed with the enantiopure compounds (*R*: 1.8 Å, PDB: 5DNX; *S*: 1.53 Å, PDB: 5DNL) were determined. These showed the same mode of mirror‐image packing as observed in *At* IGPD2 (see Figure S8), thus suggesting that the ability of IGPD to accommodate opposite chiral forms of **C348** is a general feature of the wider enzyme superfamily, rather than a peculiarity of the *Arabidopsis* enzyme. We have previously proposed that catalysis by IGPD involves conversion between an open conformation of the enzyme, which binds to imidazole‐IGP (PDB: 4MU3), and a closed conformation, which binds imidazolate–IGP (PDB: 4MU4), wherein a distinctly different binding site for the substrate–phosphate moiety is utilized.^7^ Comparison of the open and closed *At* IGPD2/substrate complexes with those of the *At* IGPD2/**C348** complexes show that neither enantiomer of **C348** can access the phosphate binding site observed in the open enzyme/substrate complex, as the backbone of the inhibitor is one carbon atom shorter than that of the substrate. Rather, both enantiomers of **C348** utilize the phosphate binding site that is associated with the closed conformation of the enzyme/substrate complex, with the ordered C‐loop; the conformation believed to be that adopted by the enzyme during catalysis, thus suggesting that both enantiomers of **C348** may mimic reaction intermediates. During the reaction catalyzed by IGPD, the adoption of an sp^2^ geometry at C3 is required for the formation of the diazafulvene intermediate, a process which is facilitated by the planar arrangement of the imidazolate ring, C3 and C2 of imidazolate–IGP. The next step in the reaction involves production of the Δ^2^‐enol, which also requires the adoption of an sp^2^ geometry at C2. This geometry necessitates that C1 moves into the plane defined by C3, C2 and the imidazolate (Figure 3 c). Comparison of the (*R*)‐ and (*S*)‐**C348** complexes with that of a modelled Δ^2^‐enol shows that the sp^2^ C2 of the Δ^2^‐enol lies in approximately the same position as that occupied by the sp^3^ C2 of both enantiomers of **C348**. Moreover, the plane defined by the main functional groups of **C348** is the same as that occupied by the modelled positions for the carbon backbone of the Δ^2^‐enol, and essentially bisects the positions of the *R* and *S* enantiomers of the inhibitor (Figure 3 d; see Figure S6b). This position implies that the two enantiomers of **C348** are equipotent nanomolar inhibitors because a) the layout of the enzyme active site facilitates mirror‐image packing and b) that they can both mimic the mode of binding of this achiral reaction intermediate.

X‐ray analysis is a powerful tool for studying the molecular basis of how ligands are recognized by biological macromolecules, but even when such studies are conducted at high resolution, ligand density can sometimes be difficult to interpret because of areas of weakness, the origins of which are often difficult to explain and are commonly cited as instances of disorder. In this study, weak electron density was observed adjacent to the chiral center of the lead compound in a 1.85 Å resolution structure of our target enzyme in complex with a racemate. By extending the resolution and chirally resolving the two enantiomers we confirmed that the areas of weakness arose from the mirror‐image packing of the two enantiomers of the inhibitor in the active site, an observation not without precedence. Without the high‐resolution data this important finding might have been overlooked in IGPD2. Significantly, the mirror‐image packing of the two enantiomers of **C348** in IGPD2 gives rise to equipotency and, as in d/l‐AOPP superinhibitors of phenylalanine ammonia‐lyase,^8^ the mimicry of a reaction intermediate gives rise to potent inhibition. This study adds to the ever‐growing body of evidence that certain enzymes are chirally promiscuous and that mirror‐image packing of ligands is a more common feature than is generally recognized in the field of drug development. Whilst the future challenge with IGPD is to exploit this understanding for the development of novel herbicides, our findings may also be relevant in other areas of drug discovery where the potential to develop inhibitors with opposite chirality may have been overlooked.

*Dedicated to the memory of Linda Britton*

## Supporting information

As a service to our authors and readers, this journal provides supporting information supplied by the authors. Such materials are peer reviewed and may be re‐organized for online delivery, but are not copy‐edited or typeset. Technical support issues arising from supporting information (other than missing files) should be addressed to the authors.

Supplementary
