# Supplementary material for: Mirror‐Image Packing Provides a Molecular Basis for the Nanomolar Equipotency of Enantiomers of an Experimental Herbicide
Source: Angew Chem Int Ed Engl. 2016 Sep 26;55(43):13485–9. doi: 10.1002/anie.201607185 (PMC5113775; doi:10.1002/anie.201607185)
Supplement: Supplementary file 1 — Supplementary [file ANIE-55-13485-s001.pdf]

Angewandte Chemie
Eine Zeitschrift der Gesellschaft Deutscher Chemiker

Supporting Information

Mirror-Image Packing Provides a Molecular Basis for the Nanomolar Equipotency of Enantiomers of an Experimental Herbicide

Claudine Bisson, K. Linda Britton, Svetlana E. Sedelnikova, H. Fiona Rodgers, Thomas C. Eadsforth, Russell C. Viner, Tim R. Hawkes, Patrick J. Baker, and David W. Rice**

anie_201607185_sm_miscellaneous_information.pdf

Supporting Information: Experimental Methods

Cloning and Protein Expression
Two constructs (A; residues 54-272, B; residues 69-272) of the gene encoding for IGPD2 (*At4g14910*) from *Arabidopsis thaliana (At)* were designed to truncate an N-terminal signal peptide from the protein according to cleavage site prediction (SignalP). Construct A was provided as a synthetic gene (GeneArt) and construct B was subsequently cloned from it and inserted into a pET24a expression vector (Novagen). The gene encoding for the full-length *Pyrococcus furiosus (Pf)* IGPD protein was PCR amplified from genomic DNA and ligated into a pETBlue vector (Novagen), before subsequently being cloned into a pET24a vector (Novagen) for protein expression. Plasmids encoding for *At* IGPD2 were transformed into *Escherichia coli* BL21(DE3) cells (Novagen) and protein expression was induced for 5 hr with 1 mM isopropyl-b-D-thiogalactopyranoside (IPTG) at 37 °C in Luria broth, supplemented with 5 mM $MnCl_2$. Plasmids encoding for the *Pf* IGPD were transformed into Tuner DE3 cells (Novagen) and expressed using the same method, but for 3 hr and supplemented with 4 mM $MnCl_2$. Cells were harvested and pellets were stored at -80 °C.

Purification
For purification, cells were defrosted and lysed by sonication. For *At* IGPD2, insoluble material was removed by centrifugation and the cell-free extract was purified in a process involving anion-exchange, hydrophobic, and size-exclusion chromatography, combined with ammonium sulfate precipitation[1]. For *Pf* IGPD, cell-free extract was heated to 70 °C for 20 min, before purification using anion-exchange and size-exclusion chromatograph. All proteins were analyzed after each purification step by SDS-PAGE. Prior to crystallization, either the racemate or resolved enantiomers of **C348** (Syngenta) were added to the protein (10 mg ml^{-1} in 50 mM Bis-Tris propane buffer pH 8.0, 50 mM NaCl, 10 mM β-mercaptoethanol, and 0.4 mM $MnCl_2$) at a final concentration of 5 mM.

Crystallisation
Initial crystallization conditions were determined by automated screening (Nextal) using a Matrix Hydra II crystallization robot. For *At* IGPD2, crystals were optimized by hanging-drop vapor diffusion using a 5:1 ratio of protein to precipitant, which yielded cubic shaped crystals after 5–10 days when equilibrated against a 1 ml reservoir of precipitant at 290K. *At* IGPD2 Crystals were cryoprotected in their crystallization solution containing 25 % – 30 % ethylene glycol, prior to flash-cooling in liquid nitrogen. The 1.1 Å complex of *At* IGPD2 with the racemate of **C348** was determined from a crystal grown from 0.1M Tris buffer pH 8.5 and 20 % PEG 550MME, whilst the complexes of *At* IGPD2 with (*R*)**-C348** or (*S*)**-C348** were determined from crystals that grew in the same pH buffer but with 20 % PEG 300 and 15 % PEG 200, respectively. All the *At* IGPD2 structures belonged to the spacegroup P432, with a cell of ~113 Å and one molecule of *At* IGPD2 in the asymmetric unit. The *Pf* IGPD crystals with (*R*)**-C348** or (*S*)**-C348** were grown from 0.1M MES buffer pH 6 and 12% or 10% MPD, respectively, using a 1:1 ratio of protein to precipitate and equilibrated against a 1ml reservoir. Diamond shape crystals grew after incubation at 290K for 7 days. Crystals were cryoprotected in their crystallization conditions plus 30% MPD and belonged to the spacegroup I422, with cell dimensions of a=b≈140.5 Å c≈137.5 Å, with three molecules of *Pf* IGPD in the asymmetric unit.

Data Collection, Processing and Structural Determination
All data sets were collected at 100K on the MX beamlines at the Diamond Light Source. Both *Pf* IGPD complexes with (*R)*- and (*S*)-**C348** and the *At* IGPD2 complex with (*R*)-**C348** were collected on beamline i04, the *At* IGPD2 complex with (*S*)-**C348** was collected on beamline i02 and *At* IGPD2 complex with the racemate of **C348** was collected on beamline i03. Data collection statistics are summarized in Table 1. Data were processed using Xia2 [2] and all additional data processing was carried out with programs from CCP4i. [3] The 1.85 Å complex of *At* IGPD2 was determined by molecular replacement using PHASER [4] with a monomer of IGPD2 from a previously determined phosphate complex (PDB: 4QNK) as a search model. The 1.1 Å complex of *At* IGPD2 with the racemate of **C348** and all subsequent *At* structures, were determined directly by refinement using the highest resolution model that was available at the time. The *Pf* IGPD complexes were determined by molecular replacement using a monomer of *At* IGPD Isoform 1 (PDB: 2F1D). [5] Model building and refinement was carried out in COOT[6] and Refmac5, [7] with ligand libraries generated by JLigand. [8] PDB ligand codes for (*R*)- and (*S*)-**C348** are 5LD and 5DL, respectively.

Refinement statistics are summarized in Table 1. For all *At* structures, no electron density was visible for the residues before S75, which was renumbered S9 for consistency with the structure of IGPD1 from *A. thaliana* (PDB: 2F1D)[5]. Models were validated using MolProbity. [9] For the *At* structures, Ramachandran values of ~95% favored, ~4% allowed and ~0.5% disallowed (R99) were observed. For the *Pf* structures ~96% were favored, ~2.5% allowed and ~1.5% disallowed (R76, D85 and R131). R99 and the equivalent residue in *Pf* IGPD, R76, are active site residues and form hydrogen bonds to the inhibitor/substrate phosph(on)ate group. D85 and R131 are within 5° of acceptable Ramachandran values, with D85 having a role in hydrogen bonding to one of the coordinating waters around Mn2 and R131 being involved in a network of salt bridges on the subunit interface. Figures were generated using Pymol. [10]

Kinetic Assays

IC50 values for the (*R*) and (*S*) enantiomers of **C348** against IGPD2 were determined by stopped assay following a previously reported protocol. [11] A 20 nM - 10μM range of inhibitor (racemate or enantiopure **C348**) was incubated with 4.76 mM IGP in assay buffer (0.1 M Hepes buffer pH 7.0 and 0.2 mM $MnCl_2$) at 30 ^{o}C for 15 mins. The reaction was started by adding 40 nM of *At* IGPD2 (frozen droplets) and left to proceed for 35 mins. The reaction was stopped by addition of 1.4 M NaOH and incubated for a further 90 mins. Absorbance was measured at 290nm and compared to a set of four pre-stopped controls, background absorbance was subtracted from all values (measured in duplicate). Fitting was carried out by nonlinear regression with equal weighting of data points using GraphPad Prism. [12] Ki was determined using the Cheng-Prusoff calculation[13] from IC50 values and the Km, which had been determined previously. [1]

References

[1] C. Bisson, K. L. Britton, S. E. Sedelnikova, H. F. Rodgers, T. C. Eadsforth, R. C. Viner, T. R. Hawkes, P. J. Baker, D.W. Rice, *Structure* **2015**, *23*, 1236-1245.

[2] G. Winter, *Journal of Applied Crystallography* **2010**, *43*, 186-190.

[3] M. D. Winn, C. C. Ballard, K. D. Cowtan, E. J. Dodson, P. Emsley, P. R. Evans, R. M. Keegan, E. B. Krissinel, A. G. Leslie, A. McCoy, S. J. McNicholas, G. N. Murshudov, N. S. Pannu, E. A. Potterton, H. R. Powell, R. J. Read, A. Vagin, K. S. Wilson, *Acta Crystallogr. Sect. D* **2011**, *67*, 235-242.

[4] A. J. McCoy, R. W. Grosse-Kunstleve, P. D. Adams, M. D. Winn, L. C. Storoni, R. J. Read, *J Appl Crystallogr* **2007**, *40*, 658-674.

[5] S. E. Glynn, P. J. Baker, S. E. Sedelnikova, C. L. Davies, T. C. Eadsforth, C. W. Levy, H. F. Rodgers, G. M. Blackburn, T. R. Hawkes, R. Viner, D. W. Rice, *Structure* **2005**, *13*, 1809-1817.

[6] P. Emsley, K. Cowtan, *Acta Crystallographica Section D-Biological Crystallography* **2004**, *60*, 2126-2132.

[7] G. N. Murshudov, A. A. Vagin, E. J. Dodson, *Acta Crystallogr D Biol Crystallogr* **1997**, *53*, 240-255.

[8] A. A. Lebedev, P. Young, M. N. Isupov, O. V. Moroz, A. A. Vagin, G. N. Murshudov, *Acta Crystallographica Section D - Biological Crystallography* **2012**, *68*, 431-440.

[9] V. B. Chen, W. B. Arendall, III, J. J. Headd, D. A. Keedy, R. M. Immormino, G. J. Kapral, L. W. Murray, J. S. Richardson, D. C. Richardson, *Acta Crystallographica Section D-Biological Crystallography* **2010**, *66*, 12-21.

[10] Schrodinger, LLC, **2010**.

[11] T. R. Hawkes, P. G. Thomas, L. S. Edwards, S. J. Rayner, K. W. Wilkinson, D. W. Rice, *Biochemical Journal* **1995**, *306*, 385-397.

[12] GraphPad Prism version 6.00 for OSX (GraphPad Software, La Jolla California USA).

[13] Y. Cheng, W. H. Prusoff, *Biochemical pharmacology* **1973**, *22*, 3099-3108.

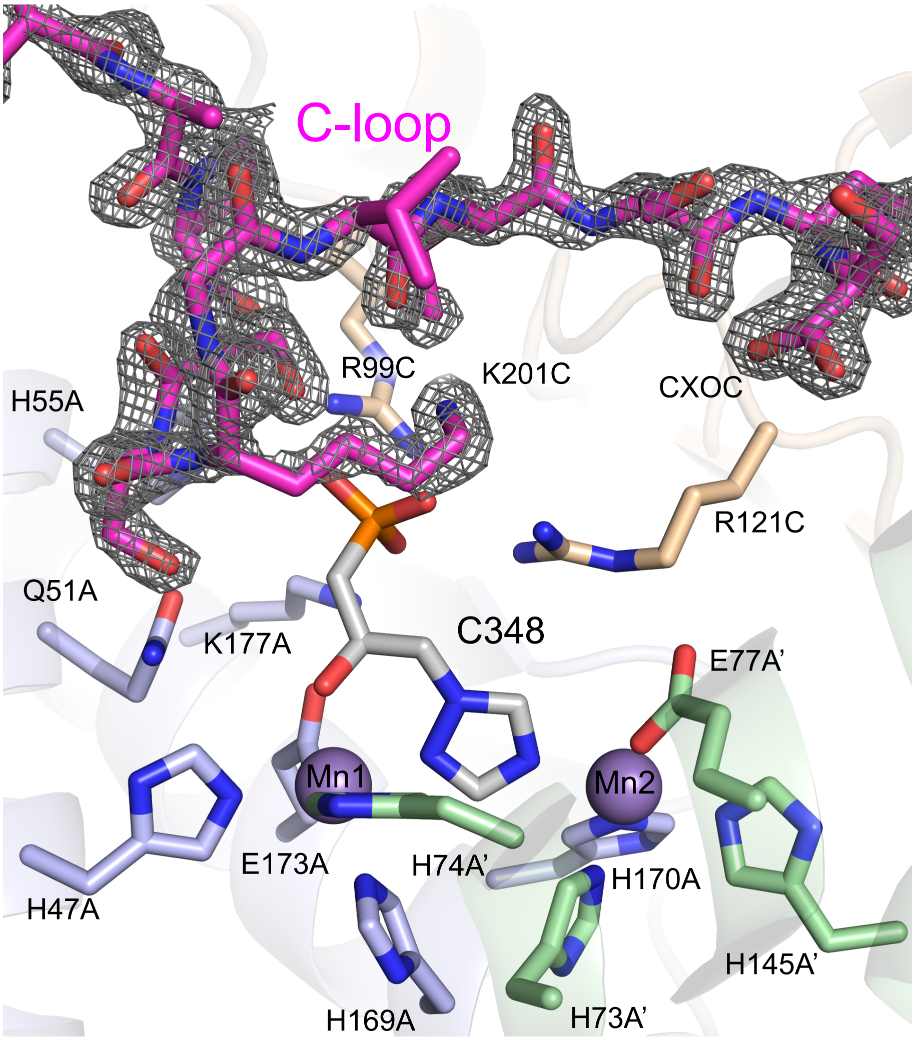

Figure S1. The position of the ordered C-loop across the face of the active site.
Cartoon representation of the C-loop (pink carbons) from the 1.85 Å IGPD2/**C348** racemate structure with the Fo-Fc electron density map (gray mesh, contoured to 3σ) derived from refining this structure with the C-loop residues omitted. Sidechains surrounding the active site are labeled and colored by chain, with a representative inhibitor drawn in gray. The two active site manganese ions are labeled and shown as purple spheres.

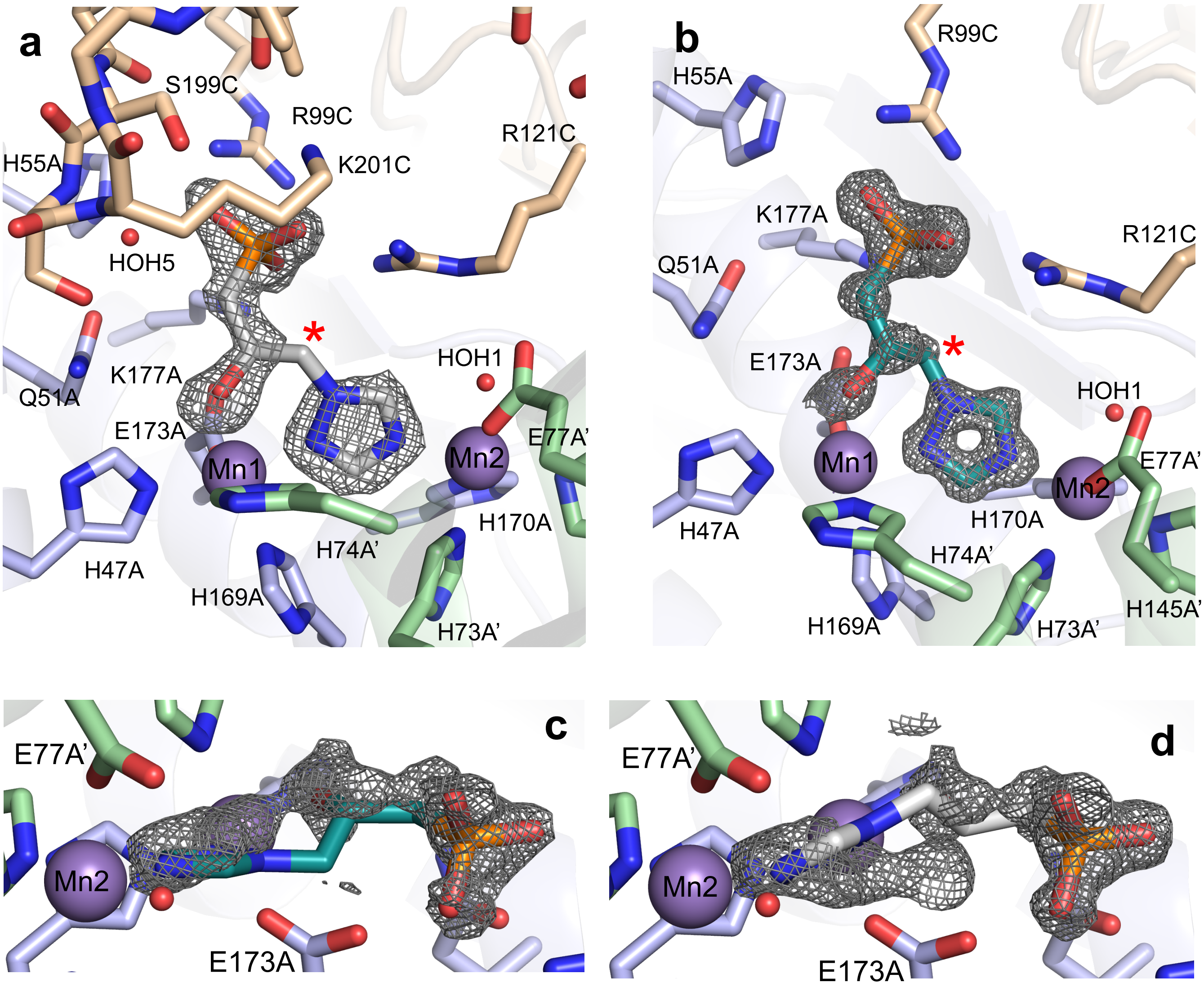

Figure S2. Discovering the binding of both enantiomers of C348 from complexes with IGPD2 (a) At 1.85 Å resolution, refinement of the protein structure, but omitting the coordinates for the ligand produced an Fo-Fc difference map that surrounds the main substituent groups of the inhibitor but with a gap in the map around the C3 atom (red asterisk) making it difficult to assign the chirality at C2 (carbon atoms in gray, *R* enantiomer shown for reference). (b) At 1.1 Å resolution, a map calculated the same way showed a clear location for the C3 atom (red asterisk), enabling the *S* enantiomer (teal) to be modeled. (c) Subsequent refinement of the 1.1 Å structure including coordinates for (*S*)-**C348,** modelled at half occupancy, produced a Fo-Fc difference map showing the clear presence of the *R* enantiomer. The binding of both enantiomers was confirmed in (d) where the 1.1 Å structure, refined with (*R*)-**C348** at half occupancy (white), showed clear difference density for the *S* enantiomer. For all parts, the Fo-Fc difference maps are drawn as a gray mesh and contoured at 3 σ. The protein is drawn as a cartoon and coloured by subunit, the sidechains are drawn as sticks with atom colours, the solvent and metal ions are drawn as red and purple spheres, respectively and in parts (b-d) the C-loop (residues 193-206) is omitted from the view for clarity.

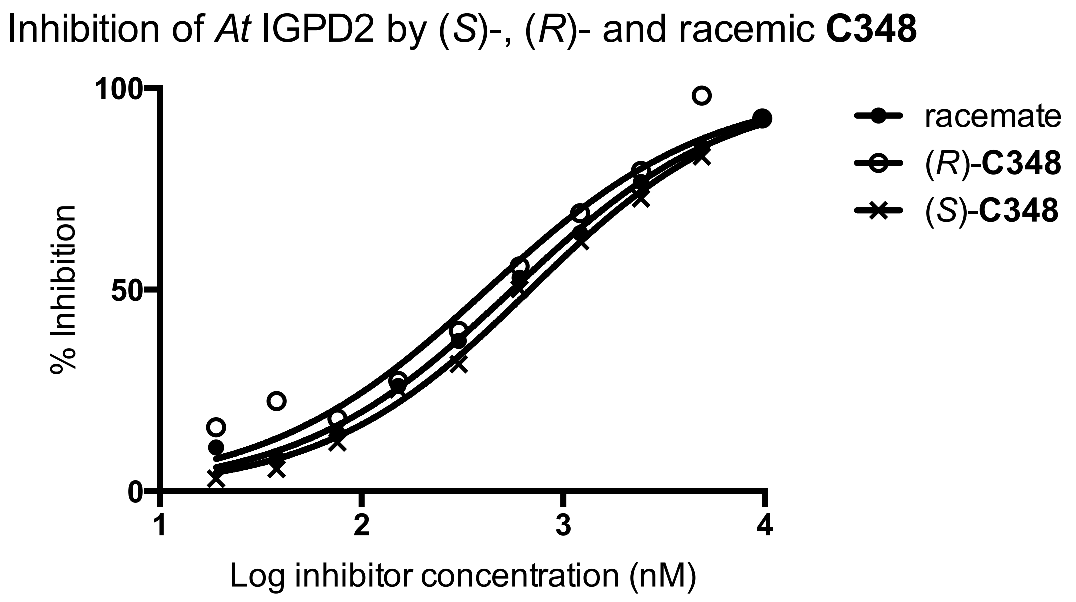

	Racemate	(*R*)-**C348**	(*S*)-**C348**
**Best-fit values**			
LogIC50	2.75	2.62	2.84
HillSlope	0.82	0.79	0.84
IC50	562	419	686
**Std. Error**			
LogIC50	0.02	0.06	0.02
HillSlope	0.03	0.08	0.04
**95% Confidence Intervals**			
LogIC50	2.70 to 2.80	2.48 to 2.77	2.78 to 2.90
HillSlope	0.74 to 0.89	0.59 to 0.98	0.75 to 0.93
IC50	502 to 629	302 to 581	606 to 777
Km IGPD2 (mM) =	0.17		
[S] (mM) =	4.76		
**Ki values (nM)**	18.9	14.1	23.0
95% confidence intervals	16.8-21.1	10.1-19.5	20.3-26.1

Figure S3. Expanded *in vitro* enzyme assay results.
In vitro Inhibition data for (*R*)-**C348**, (*S*)-**C348** and the racemate fit to log IC50 values of 2.62, 2.84 and 2.75 (95% confidence limits ± log 0.15, Hill coefficients of ~0.8) corresponding to Ki values of ~14±4, 23±3 and 19±3 nM, respectively. Substrate IGP concentration was 4.76 mM and the Km for IGP was 170 μM. Fitting was carried out by nonlinear regression with equal weighting of data points using GraphPad Prism. [12]

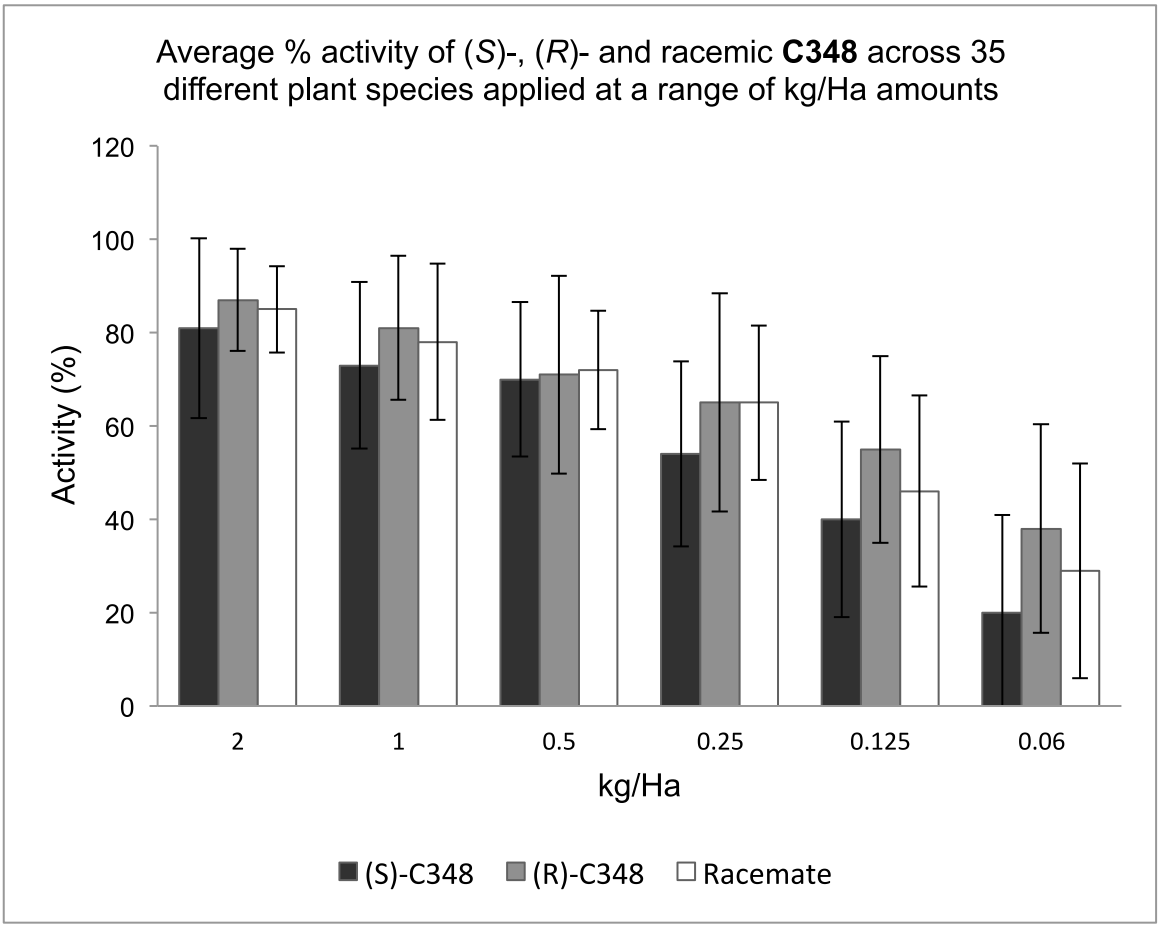

Figure S4. *In vivo* response to C348.
Average % activity of (*S*)-**C348** (black), (*R*)-**C348** (gray) and the **C348** racemate (white) at a range of kg/Ha amounts. Activity was estimated by average visual damage scores (0-100%) assessed 14 days after spray treatment of glass house of soil-grown 1-2 week old seedlings of 35 plant species including broad-leaved and grass crops, as well as weeds from both cool and warm climate regions. Glass-house herbicide spray treatment, adjuvancy and assessment methods were as standard in the agrochemical industry. The standard deviation is shown for each data set.

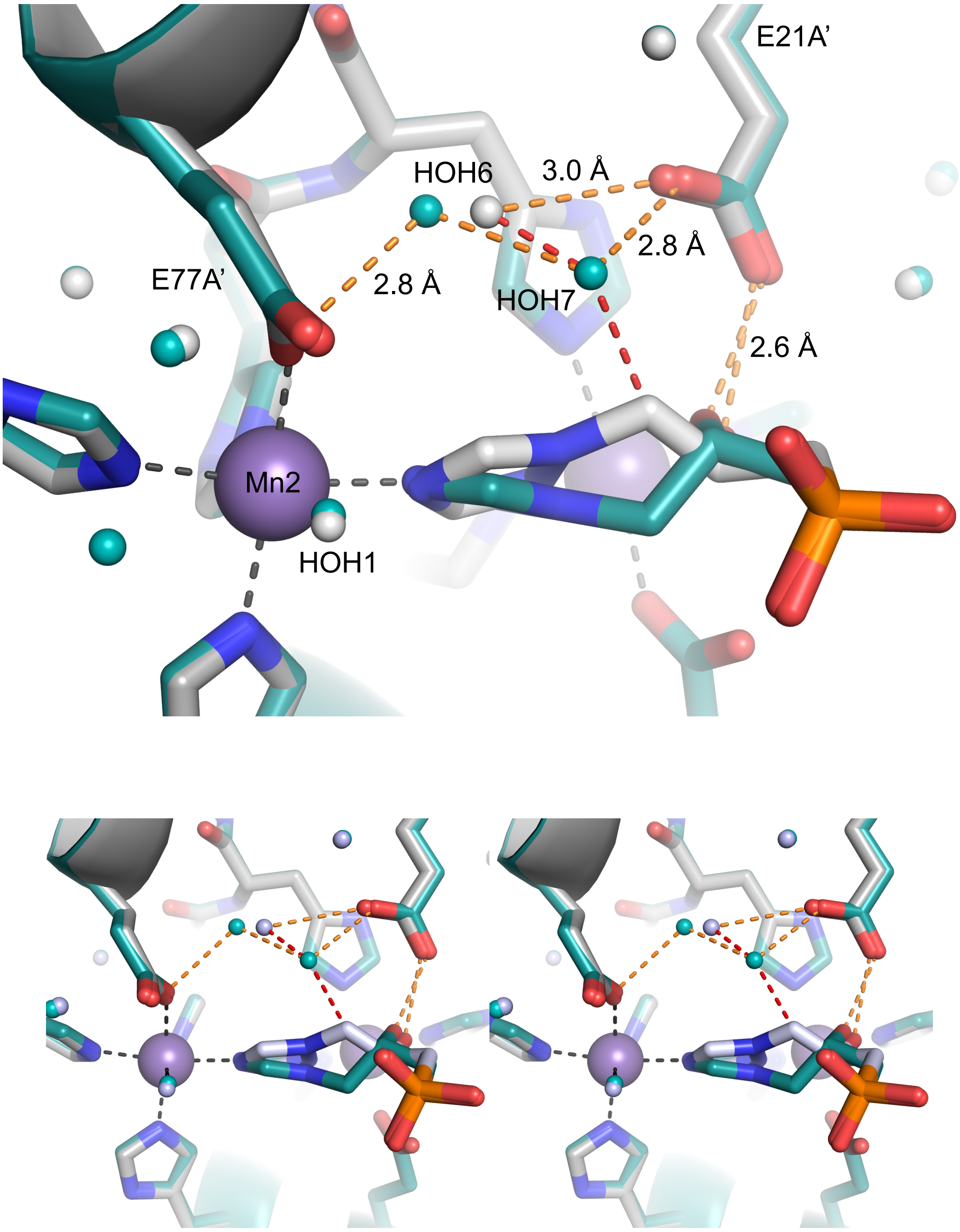

Figure S5. Comparison of the solvent structure within the active site of the enantiopure (*R*)-C348 and (*S*)-C348 complexes with IGPD2.

In the (*S*)**-C348** complex (teal), two ordered water molecules (HOH6 and HOH7) are located within the active site, forming hydrogen bonds with side chain oxygen atoms from E21 and E77. In the structure of the (*R*)**-C348** complex (white), HOH7 is absent due to adverse steric interactions that would occur with the C3 atom of the inhibitor, and resulting in a concomitant shift in the position of HOH6 by approximately 0.8 Å. Important amino acid side chains, water molecules and ligands for (*R*)-**C348** and (*S*)-**C348** are labeled, gray dashed lines indicate metal-ligand contacts, orange dashed lines indicate hydrogen bonds and red dashed lines indicate steric clashes. Mn2 is labeled and drawn as a purple sphere. A stereo view is also shown.

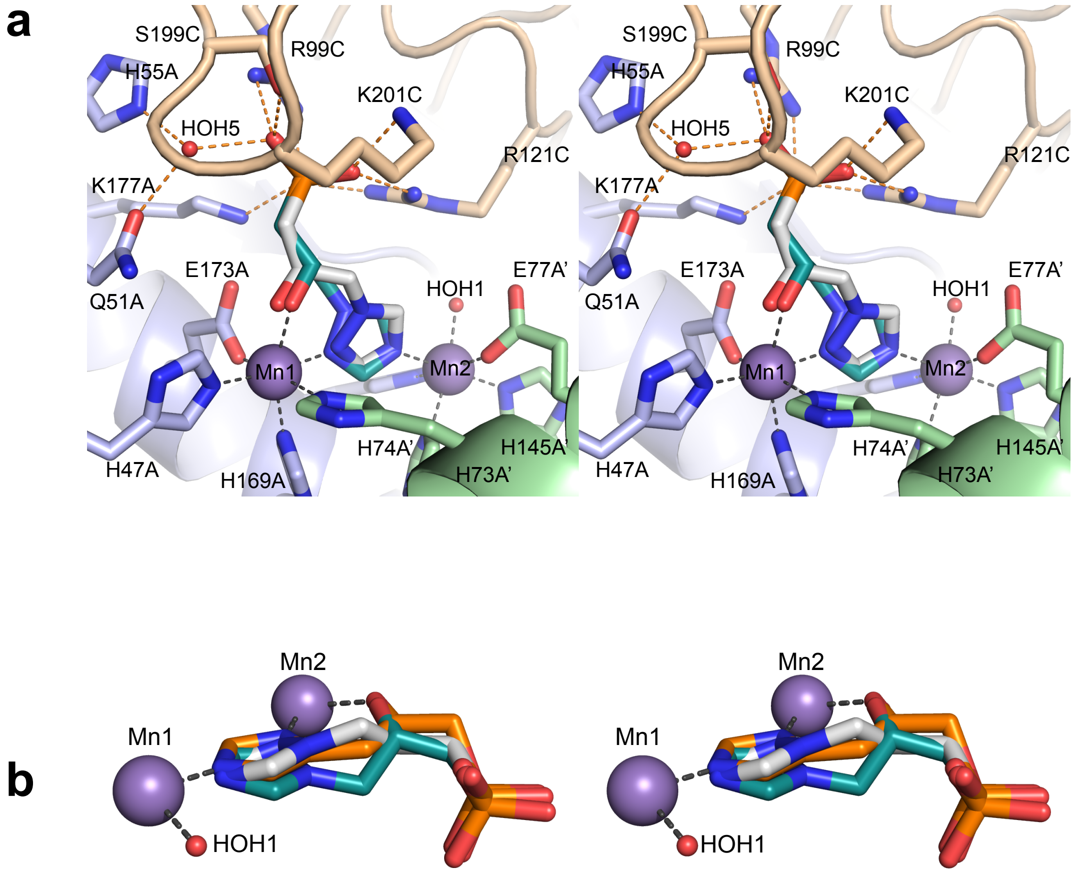

Figure S6. Stereo views of superpositions of the (*S*)- and (*R*)-C348 complexes with IGPD2 and a comparison of the C348 enantiomers with the modelled Δ^2-enol reaction intermediate
(a) The protein is drawn as a cartoon and coloured by subunit, the sidechains are drawn as sticks with atom colours and labeled and with the solvent and metal ions drawn as red and purple spheres, respectively. The *R* enantiomer of **C348** is coloured with white carbons and the *S* enantiomer is coloured with teal carbons. In (b) the two enantiomers are superimposed onto the position of a modelled Δ^2-enol intermediate

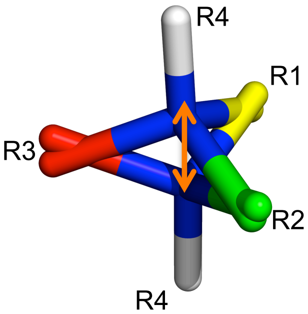

D=distance between the two chiral centres

R4 is commonly a hydrogen.

Enzyme/diagram of ligand	D (Å)	d(min) (Å)	PDB
Imidazoleglycerol-phosphate dehydratase (IGPD) 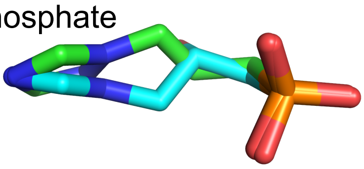	0.4	1.1/1.4 or 1.5/1.8	5ELW 5EL9 or 5DNX 5DNL
Alanine racemase 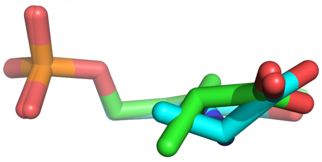	1	2.0/2.0	1L6F 1L6G
Burkholderia cepacia lipase 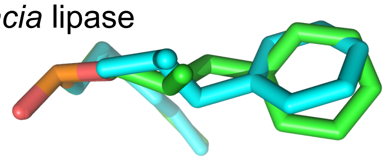	0.8	1.1/1.5	1YS1 1YS2
Human retinoic acid receptor 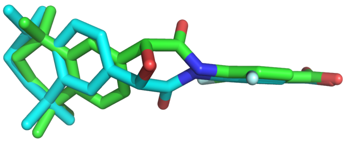	1.5	1.6/1.7	1EXA 1EXX
Inositol monophosphatase 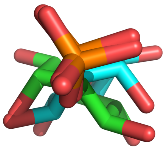	1.3 1.7	2.3/2.2	1IMA 1IMB
Citrate synthase 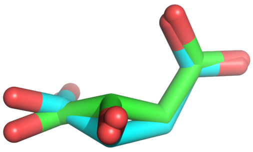	0.7	1.9/1.9	3CSC 4CSC
Isocitrate dehydrogenase 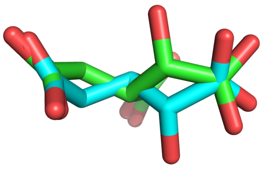	0.4	1.7/1.85	1PB1 1P8F

Figure S7. Examples of mirror-image packing. A selection of proteins are listed, with the opposite chiral forms of the bound ligand (coloured cyan and green) overlaid. The distance between the chiral centre in each pair of enantiomers is noted as D and indicated in the top schematic with an orange double headed arrow. The resolution of the structures and PDB codes are listed. References can be found in the main text.

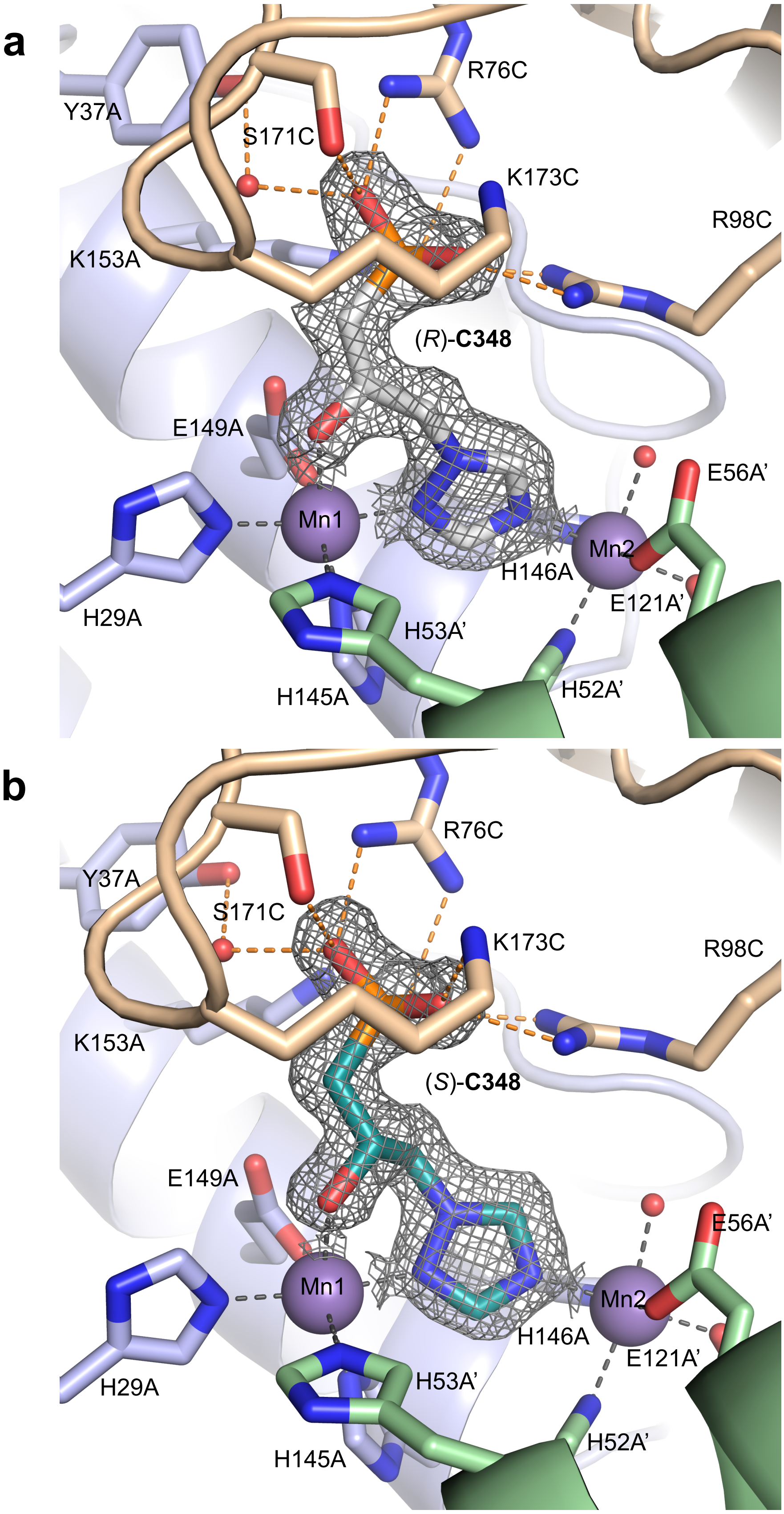

Figure S8. Enantiopure (*R*)-C348 and (*S*)-C348 complexes with IGPD from *Pyrococcus furiosus*.

Cartoon representation of the inhibitor bound within the active site with the corresponding Fo-Fc electron density map (gray mesh, contoured at 3σ) calculated with the inhibitor omitted from the model. Important sidechains surrounding the active site are labeled and colored by chain, the two active site manganese ions are labeled and shown as purple spheres and important water molecules are drawn as smaller red spheres. Grey dashed lines indicate metal-ligand contacts and orange dashed lines indicate hydrogen bonds

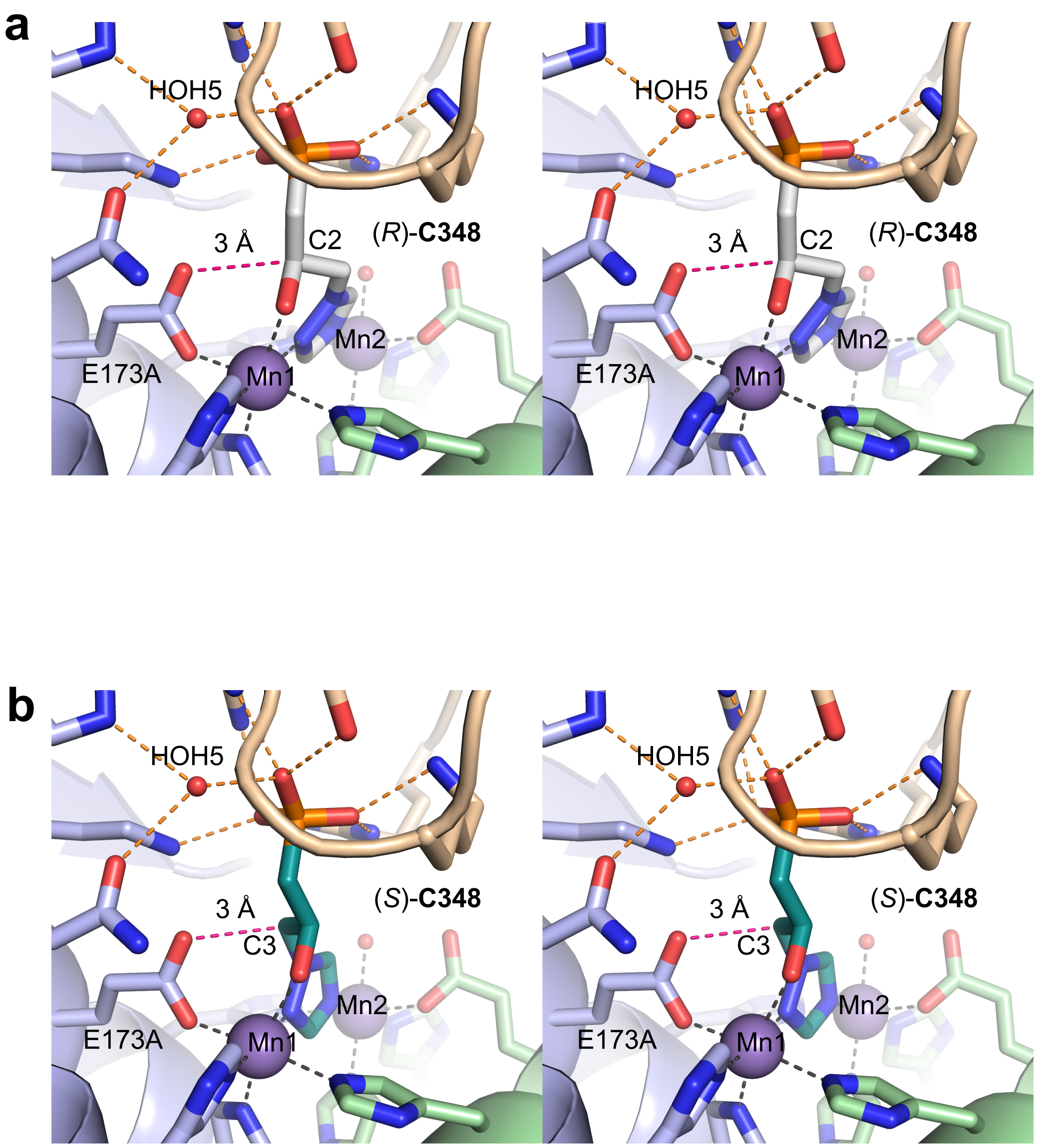

Figure S9. Stereo views showing the presence of C-H…O hydrogen bonds in structures of IGPD2 in complex with enantiopure (*R*)-C348 and (*S*)-C348.
(a) The 1.5 Å (*R*)-**C348** (white) complex with IGPD2 showing the close approach (pink dashed line) of the (*R*)-**C348** C2 to OE2 of E173 from chain A. (b) The 1.15 Å (*S*)-**C348** (teal) complex with IGPD2 showing the close approach (pink dashed line) of the (*S*)-**C348** C3 to OE2 of E173 from chain A, indicating in both cases that a C-H…O hydrogen bond is present implying that E173 is basic. Important sidechains surrounding the active site are labeled and colored by chain, the two active site manganese ions are labeled and shown as purple spheres and important water molecules are drawn as smaller red spheres. Gray dashed lines indicate metal-ligand contacts and orange dashed lines indicate hydrogen bonds.
